# A New Brazilian Device for Cervical Cancer Screening: Acceptability and Accuracy of Self-sampling

**DOI:** 10.1055/s-0043-1770134

**Published:** 2023-06-20

**Authors:** Martina Lichtenfels, Noely Paula Cristina Lorenzi, Maricy Tacla, Kaori Yokochi, Flávia Frustockl, Camila Alves Silva, André Luiz da Silva, Lara Termini, Caroline Brunetto Farias

**Affiliations:** 1 Ziel Biosciences, Porto Alegre, RS, Brazil; 2 Department of Gynecology, Instituto do Câncer do Estado de São Paulo, São Paulo, SP, Brazil; 3 Department of Gynecology, Hospital das Clínicas, Faculdade de Medicina, Universidade de São Paulo, São Paulo, SP, Brazil; 4 Citoclin Laboratory, Porto Alegre, RS, Brazil; 5 Santa Casa de Misericórdia de Porto Alegre, Universidade Federal de Ciências da Saúde de Porto Alegre, Porto Alegre, RS, Brazil; 6 Centro de Investigação Translacional em Oncologia, Instituto do Câncer do Estado de São Paulo, Hospital das Clínicas, Faculdade de Medicina da Universidade de São Paulo, São Paulo, São Paulo, Brazil

**Keywords:** uterine cervical neoplasms, human papillomavirus, cytology, diagnostic screening programs, early detection of cancer, Neoplasias do colo de útero, Papiloma vírus humano, Programas de rastreamento, Detecção precoce do câncer

## Abstract

**Objective** To evaluate the accuracy and patient acceptability toward self-sampling using a new device - SelfCervix® - for detecting HPV-DNA.

**Methods** A total of 73 women aged 25–65 who underwent regular cervical cancer screening from March to October 2016 were included. Women performed self-sampling followed by a physician-sampling, and the samples were analyzed for HPV-DNA. After that, patients were surveyed about their acceptability of self-sampling.

**Results** HPV-DNA detection rate of self-sampling presented high accuracy and was similar to physician-collection. Sixty-four (87.7%) patients answered the acceptability survey. Most patients (89%) considered the self-sampling comfortable, and 82.5% preferred self-sampling to physician-sampling. The reasons cited were time-saving and convenience. Fifty-one (79.7%) reported that they would recommend self-sampling.

**Conclusion** Self-sampling using the new Brazilian device SelfCervix® is not inferior in HPV-DNA detection rate compared with physician-collection, and patients are supportive of the method. Therefore, it might be an option to reach under-screened populations in Brazil.

## Resumo

**Objetivo** Avaliar a acurácia e aceitabilidade da auto-coleta utilizando um novo coletor - SelfCervix® - para a detecção de DNA de HPV.

**Métodos** Foram incluídas no estudo 73 mulheres com idade entre 25–65 anos que realizaram seu rastreamento regular do câncer de colo do útero entre Março e Outubro de 2016. Estas mulheres realizaram a auto-coleta, seguida de coleta profissional e as amostras foram analisadas para a presença de DNA de HPV. Após, elas responderam um questionário sobre a experiência da auto-coleta.

**Resultados** As taxas de detecção de DNA de HPV por auto-coleta foram altas e similares as da coleta profissional. Sessenta e quatro (87,7%) pacientes responderam o questionário de experiência. A maioria (89%) considerou a auto-coleta confortável, e 82,5% preferiram o método comparado a coleta profissional. As razões citadas foram economia de tempo e conveniência. Cinquenta e uma (79,7%) mulheres confirmaram que recomendariam a auto-coleta.

**Conclusão** Auto-coleta utilizando o novo coletor desenvolvido no Brasil não é inferior na detecção de DNA de HPV quando comparada a coleta profissional, e apresenta uma boa aceitabilidade pelas mulheres. Desta maneira, pode ser uma opção para alcançar populações que não realizam o rastreamento padrão.

## Introduction

Cervical cancer is the fourth most common cancer in women worldwide and is responsible for ∼311,000 deaths per year. 1 Despite highly preventable neoplasia, this tumor frequently occurs in women who do not participate in screening programs 2 3 Papanicolaou (Pap) test is Brazil's gold-standard method for cervical cancer screening and since the introduction of screening programs, the early diagnosis has decreased considerably the cervical cancer burden. 4 In Brazil, Pap testing-based cervical cancer screening programs are available for the population through the public health system, however, many women do not attend the programs and are not reached by them. Problems such as lack of knowledge, physician embarrassment, competing priorities, and access difficulties to the public health system are associated with not-attendance to screening programs. 5 Therefore, the adoption of alternative methods to complement the traditional screening already available is needed.

Persistent infection with high-risk Human Papillomavirus (mainly HPV types 16 and 18) is the etiologic cause of cervical cancer development. 6 This link between HPV infection and cervical cancer supported the introduction of HPV testing in screening programs. 7 8 The HPV testing presented high negative predictive results and is very sensitive for detecting patients at high risk of developing cervical cancer precursor lesions and cancer. 8 9 10

Self-sampling of cervicovaginal specimens is feasible, can be done at a convenient location and time, is cost-effective, avoids the need for a professional-based sampling, and enhances women's empowerment for their health. The combination of self-sampling with HPV DNA testing has similar accuracy compared with professional-based collection, 11 12 can increase screening adherence in populations under-screened, 9 13 14 and is a promising strategy for expanding screening coverage.

We developed a Brazilian self-collector of cervicovaginal samples, SelfCervix®, to collect enough cells to perform HPV DNA testing, liquid-based cytology, and analysis of several sexually transmitted diseases. Our study aims to evaluate self-sampling acceptability and accuracy using the SelfCervix® for detecting HPV DNA in a Brazilian cohort.

## Methods

Women aged 25–65 years who underwent previous Pap testing for cervical cancer screening at USP Clinic Hospital (São Paulo, SP, Brazil), USP University Hospital (São Paulo, SP, Brazil), and Citoclin (Porto Alegre, RS, Brazil) from March to October 2016 were invited to participate in the study. Pregnant women, women who have not yet started the sexual activity, or those who underwent chemotherapy or radiotherapy for cervical cancer were not eligible for the study. Patients presented the following results in the previous Pap testing: no histology alteration in one (1.6%) sample, cervicitis in 13 samples (20.2%), atypia in one (1.6%) sample, Cervical intraepithelial neoplasia I (CINI) in 13 (20.2%) samples, CIN2/CIN3 in 31 (48.4%) samples, adenocarcinoma in two (3.2%) samples, condyloma/warts in one (1.6%) sample, and squamous metaplasia in two (3.2%) samples. From 9 patients, the histology information was missing due to insufficient samples to perform the test.

Women who provided informed consent performed self-sampling followed by a physician-sampling with a vaginal swab. Before the physician-sampling, the SelfCervix® and verbal instructions explaining how to carry out the cervical self-sampling were provided to each patient by the physician. The SelfCervix® comprises a plunger with a soft material in the distal portion. After inserting the device into the vagina, women depressed the plunger, and through rotational movements, the device collected the cells from the cervicovaginal area. Once collected, the material was placed in a tube containing PreservCyt® (Hologic, MA, USA) medium. The material obtained was used for carrying out HPV-DNA tests and liquid-based cytology when the HPV-DNA was positive.

After the self-sampling using SelfCervix®, the physician collected cervical smears with a vaginal swab, and the patient underwent a standard professional exam. The sample was placed in a tube containing PreservCyt® (Hologic, MA, USA) medium for carrying out HPV-DNA test and liquid-based cytology. Our study's gold-standard analysis of cervical samples was the HPV-DNA detection rate by physician-based sampling. The samples were classified according to the International Federation for Cervical Pathology and colposcopy 2011. 15

The HPV-DNA test was performed using the hybrid capture technique II Qiagen (Gaithersburg, USA). Oncogenic probes were used to identify HPV 16, 18, 31, 33, 35, 39, 45, 51, 52, 56, 58, 59, and 68. For HPV-DNA-positive women, liquid-based cytology was performed on the same cervical sample.

When the cytology was positive for any cervical abnormality (diagnosis of atypical cells of undetermined significance or more (ASCUS + )), the woman was referred for colposcopy and classified according to Bethesda classification: CIN 1 corresponds to low-grade squamous intraepithelial neoplasia and CIN 2 and 3 to high-grade squamous intraepithelial neoplasia.

The HPV-DNA testing, liquid-based cytology, and colposcopy were analyzed at the Gynecological Oncology Research Institute (IPOG) laboratory based in São Paulo.

The participants were invited to complete the acceptability of vaginal self-sampling survey following the self and physician-sampling. The survey consisted of 15 questions regarding sexual activity, prior experience with Pap testing, tolerability of both methods, preferences for self or physician-sampling, reasons for preferring one approach, and if they will indicate the self-sampling for other women.

Continuous variables were presented as mean and standard deviation and frequency and percentage for categorical variables. The comparison between self-sampling and physician collection samples was analyzed using the Kappa index. Value 0 was considered poor agreement, between 0 - 0.2 reasonable agreement, 0.2–0.4 agreement, 0.4–0.6 moderate agreement, 0.6–0.8 substantial agreement, and above 0.8 excellent agreement. Fischer's exact test and chi-square were used to assess the relationship between each technique and colposcopy and biopsy results. The SPSS version 19.0 (IBM Inc, Chicago, IL) was used. A p-value < 0.05 was considered significant with a 95% confidence interval.

This study was performed according to The Code of Ethics of the World Medical Association (Declaration of Helsinki) ethical guidelines and was approved by the ethics committee of the USP University Hospital under the number CAAE 56311616.6.0000.0076 (approval date: 24 June 2016) and USP Clinic Hospital CAEE 38719314.2.0000.0068 (approval date: 11 April 2017). All patients signed the informed consent form to participate in the study.

## Results

A total of 73 women from 3 different institutions were included in our study. Twenty-two (30.1%) women underwent cervical sample collection at Citoclin, 17 (23.3%) at the University Hospital of São Paulo (HU), and 34 (46.6%) at Clinic Hospital of São Paulo (HC). The median age of the patients was 33 years.

**Fig. 1:**
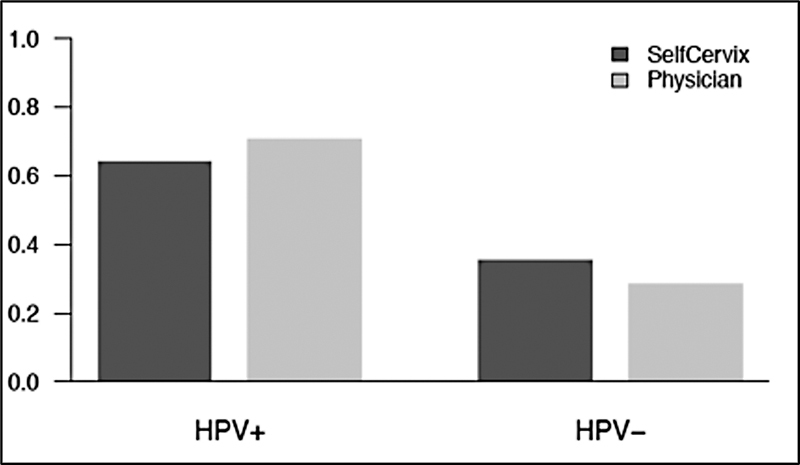
The detection rate of HPV-DNA in cervical samples collected by self-sampling and physician-sampling.

The detection rate of HPV-DNA in self-collected and physician-collected samples was 64.4% and 71.2%, respectively ( *p* = 0.1) ( Fig. 1 ).

Discrepancies in HPV-DNA detection between self and physician-collected samples occurred in 9 (12.3%) cases. Of these samples, 7 were positive for HPV-DNA as determined in the physician-sampling and the other 2 samples corresponded to cases classified as negative for HPV-DNA in physician-sampling and positive in self-sampling. All HPV-DNA-positive samples in physician and self-sampling were referred to liquid-based cytology. In the discordant results, the physician-sampling was the gold-standard for liquid-based cytology. If the sample was positive for HPV in physician-sampling and negative in self-sampling, only the physician-sampling was referred to liquid-based cytology; and if the sample was HPV negative in physician-sampling but positive in self-sampling, both samples were referred to liquid-based cytology. “These results are summarized in table 1 .

**Table 1:** Characteristics of discordant results in samples collected by self-sampling and physician-sampling

		Physician-sampling		Self-sampling	
Age	Histology	HPV	Liquid-based cytology	HPV	Liquid-based cytology
30	Cervicitis	+	Inflammation	−	Not performed
32	CIN2/CIN3	+	ASCUS	−	Not performed
36	CIN2/CIN3	+	ASCUS	−	Not performed
45	Cervicitis	+	ASCUS	−	Not performed
35	CIN2/CIN3	+	Low grade	−	Not performed
27	CIN2/CIN3	+	High grade	−	Not performed
24	Missing data	+	High grade	−	Not performed
22	Cervicitis	−	Inflammation	+	Inflammation
23	Cervicitis	−	Inflammation	+	Inflammation

Abbreviations: ASCUS, Atypical squamous cells of undetermined significance; CIN, Cervical intraepithelial neoplasia; HPV, Human Papillomavirus.

In our study self-sampling presented 87% accuracy, 86% sensibility, and 84% efficiency for HPV-DNA detection. For HPV-DNA-positive women, liquid-based cytology was performed on the same cervical sample to analyze cellular alterations and also presented 87% accuracy. The HPV-DNA detection rate of the samples collected by the physician was used as the gold standard for comparisons ( Table 2 ).

**Table 2:** Efficacy of self-sampling for HPV-DNA detection compared with physician-sampling physician-collection (gold-standard)

	HPV
Sensibility	0.86
NPV	0.95
PPV	0.73
Specificity	0.90
Accuracy	0.87
Efficiency	0.84

Abbreviations: HPV, Human Papillomavirus; NPV, Negative predictive value; PPV, Positive predictive value.

Of 73 patients, 64 (87.7%) answered the survey. The mean age was 35 (18–65) years old. Approximately half of the patients, 31 (48.4%) started sexual activity between 18 and 20 years, and 38 (59.3%) did not use a condom in sexual relationships. All the patients underwent prior Pap testing at least once in life, 71.4% perform the screening annually, and 22.2% every three years. Fifty-seven patients (89%) considered self-sampling tolerable/painless, and 29 (45.3%) reported self-sampling was more comfortable to use than physician-sampling. The majority (82.5%) preferred self-sampling to physician-sampling, and the reasons cited were convenience (54.7%) and time-saving (30%). Most patients (79.7%) reported that they would recommend self-sampling to other women.

## Discussion

To our knowledge, this is the first Brazilian self-collector product, SelfCervix® (ANVISA registry 80525329009), that allows the analysis of HPV and liquid-based cytology with a unique collection.

HPV testing as a primary screening method for cervical lesions and cancer is approved in different countries, including the USA, England, and the Netherlands. 16 17 18 HPV-based cervical cancer screening increased by 90% the detection of CIN3+ and, due to the high negative predictive value, is considered superior to cytology. 16 19 20 A population-based study with 1.160,981 women from rural China demonstrated the efficiency of HPV testing in detecting CIN2+ lesions and supports the introduction of HPV testing in primary screening in China. 21 The benefit of HPV DNA testing for cervical cancer screening is still debatable regarding the detection of adenocarcinomas. HPV testing is less sensitive for adenocarcinoma precursors compared with squamous cancer precursors. 22 23 However, since none of the available screening options, cytology and HPV testing, are able to detect all cervical cancer, the HPV testing is presenting better results for the detection of overall cervical carcinoma and precursors. 16 23 24 In a preliminary analysis, a study conducted in 2017 in Indaiatuba city (SP, Brazil) evidenced increased coverage, high adhesion to follow-up, few unsatisfactory samples, and a high referral for colposcopy using DNA-HPV testing as a primary screening program compared with cytology. 25 26 In population-based data from Brazil, cervical cancer screening with HPV testing was also cost-effective compared with cytology. 27 Indeed, screening every 4 years using HPV testing presented a lower cost. 28 Current study showed a probability lower than 1% of CIN2+ detection ten years after a negative HPV test and suggested that the interval of HPV testing could be prolonged in selected women. 29 Despite that, until now in Brazil the gold-standard method for cervical cancer screening is the Papanicolaou (Pap) test, and HPV DNA testing is available on public health system only for sexual disease diagnosis.

In the present study, we demonstrated that self-sampling of cervicovaginal samples using the SelfCervix® presented high accuracy, sensibility, and good specificity to detect HPV-DNA and cellular alterations in liquid-based cytology for positive HPV-DNA samples. Corroborating our findings, previous studies presented similar HPV-DNA detection accuracy on self and physician-based sampling, good acceptability, and preference for self-sampling in populations under-screened and women attending routine cervical screening. 9 13 30 31 32 33 Lorenzi and colleagues evaluated the acceptability of cervicovaginal self-sampling in a cross-sectional study with 116 women from two university hospitals in Brazil. The authors demonstrated that most women preferred self-sampling to the collection by a healthcare professional due to the possibility of choosing the place and the best time to perform the sampling. 34 Castle and colleagues showed a preference for under-screened Brazilian women to perform self-sampling. The authors suggested that self-sampling combined with HPV-DNA testing could improve screening coverage in Brazil and reach women who do not have access to the Pap testing. 9 Torres and colleagues also showed that cervicovaginal self-collection with detection of cervical malignancy using HPV 16 and 18 E6 oncoproteins is feasible and expanded screening coverage in women from a remote geographic location in Amazonas (Brazil). 14 In Argentina, the self-collection with HPV testing increased 4-fold the cervical screening coverage through a community of health workers. 13 The increased coverage makes HPV testing with self-collection most cost-effective than traditional screening methods in low and middle-income countries. 32 Future studies using SelfCervix® in HPV-based cervical cancer screening in a large cohort must verify the acceptability, coverage, and cost-effectiveness.

We observed an HPV detection concordance of 87.7% between the SelfCervix® and physician-based sampling. In accordance, previous authors showed over 90% of HPV detection concordance between self and clinician-collected samples. 12 35 36 Nine women presented discordant results in HPV-DNA testing: in the self-sampling, two samples were positive, and seven were negative, contrary to professional-sampling findings. It is interesting to highlight that the patients with discordant results were younger, with a median age of 29.5 years and none of the patients aged > 45 years presented disagreement in HPV results with the different collection types. The patient’s age and HPV testing are important factors to be considered when evaluating HPV self-sampling.

We compared the results of a meta-analysis already published in the literature with the results of a Pap testing collected by a health professional to our findings of HPV-DNA detection rate by the SelfCervix® device. The Selfcervix® presented a sensitivity of 86% to detect HPV-DNA compared with the meta-analysis results that demonstrated 59% sensitivity of the Pap testing for cervical cancer screening strategy. The two methodologies showed a similar specificity (90% versus 94%). 37 When comparing data already published from other cervical collectors 34 to the SelfCervix®, we observed that SelfCervix® presented a tendency of higher sensibility (86% versus 74%) and similar specificity (90% versus 92%) in HPV detection. The studies evaluating other cervical collectors used HC2 assay to detect HPV-DNA (16,18,31,33,35,39,45,51,52,56,68,69 and 68) and swab, spatula/Cytobrush, cervix broom brush, and Digene sampler for cervicovaginal sampling. 37

We also performed a survey with the participants to evaluate women's perception of the use of self-sampling. They reported a preference for self-sampling compared with physician-sampling, considering convenience the most important perceived benefit, and almost 80% would recommend it. Nine women did not answer the survey claiming a lack of time. These findings follow previous literature showing positive feedback from women and good acceptability of self-sampling. 9 34 38 39 A literature review including articles from low-and middle-income countries showed that most patients considered HPV self-sampling easy to perform, painless and preferred compared with physician-sampling. The most reported benefits were the convenience of screening from home, time-saving and less embarrassment. 38 Highly acceptability of HPV self-sampling, regardless of age and country of residence, and a preference for home-based self-sampling was also evidenced in a systematic review comprising 72 studies published between 2002 and 2018. 39 Lorenzi and colleagues evaluated the acceptability of self-sampling in a cross-sectional study involving 116 Brazilian women. The authors showed that most participants considered self-sampling easy to collect cervicovaginal samples and preferred self-sampling over physician-collection. Corroborating our results, the participants also reported the convenience of choosing the place and time for sampling as the main benefit. 34

Our study presented limitations, including the sample size, the possible difference in the professional-sampling and cytology evaluation from the 3 different institutions, and the use of professional-sampling as the gold-standard methodology instead of anatomo-pathological analysis.

## Conclusion

The self-collector of cervicovaginal samples, SelfCervix®, demonstrated high performance in HPV-DNA detection and patient acceptability in a Brazilian cohort. The SelfCervix® is not inferior in the detection of HPV compared with physician-collection. We suggest that the use of SelfCervix® in combination with HPV testing might be an option to reach under-screened populations and increase the coverage of cervical cancer screening in Brazil.
